# Multiple Immune Features-Based Signature for Predicting Recurrence and Survival of Inoperable LA-NSCLC Patients

**DOI:** 10.3389/fonc.2020.571380

**Published:** 2020-10-14

**Authors:** Meiying Guo, Wanlong Li, Butuo Li, Bing Zou, Shijiang Wang, Bingjie Fan, Xindong Sun, Linlin Wang

**Affiliations:** ^1^Department of Radiation Oncology, Qilu Hospital of Shandong University, Jinan, China; ^2^Shandong Cancer Hospital and Institute, Shandong First Medical University and Shandong Academy of Medical Sciences, Jinan, China; ^3^Key Laboratory of Cancer Prevention and Therapy, Department of Radiation Oncology, National Clinical Research Center for Cancer, Tianjin Medical University Cancer Institute and Hospital, Tianjin, China

**Keywords:** immune signature, locally advanced non-small cell lung cancer, least absolute shrinkage and selection operator, nomogram, programmed death ligand 1, CD8

## Abstract

**Introduction:**

The immune status of the tumor microenvironment is extremely complex. One single immune feature cannot reflect the integral immune status, and its prognostic value was limited. We postulated that the immune signature based on multiple immuno-features could markedly improve the prediction of post-chemoradiotherapeutic survival in inoperable locally advanced non-small-cell lung cancer (LA-NSCLC) patients.

**Methods:**

In this study, 100 patients who were diagnosed as having inoperable LA-NSCLC between January 2005 and January 2016 were analyzed. A five immune features-based signature was then constructed using the nested repeat 10-fold cross validation with least absolute shrinkage and selection operator (LASSO) Cox regression model. Nomograms were then established for predicting prognosis.

**Results:**

The immune signature combining five immuno-features was significantly associated with overall survival (OS) and progression-free survival (PFS) (*P* = 0.002 and *P* = 0.014, respectively) in patients with inoperable LA-NSCLC, and at a cutoff of −0.05 stratified patients into two groups with 5-year OS rates of 39.8 and 8.8%, and 2-year PFS rates of 22.2 and 5.5% for the high- and low-immune signature groups, respectively. Integrating immune signature, we proposed predictive nomograms that were better than the traditional TNM staging system in terms of discriminating ability (OS: 0.692 vs. 0.588; PFS: 0.672 vs. 0.586, respectively) or net weight classification (OS: 32.96%; PFS: 9.22%), suggesting that the immune signature plays a significant role in improving the prognostic value.

**Conclusion:**

Multiple immune features-based immune signature could effectively predict recurrence and survival of inoperable LA-NSCLC patients and complemented the prognostic value of the TNM staging system.

## Introduction

Lung cancer remains the leading cause of death worldwide, and approximately 30% of non-small-cell lung cancer (NSCLC) patients are diagnosed with locally advanced disease (LA-NSCLC) (1, 2). Most LA-NSCLC patients are unable to undergo surgery, and platinum-based chemotherapy combined with concurrent thoracic radiotherapy is the first-line treatment for this group of patients (3). However, their prognosis is quite different, with a 2-year overall survival (OS) rate of 24%–55% and a 5-year OS rate of 13–36% (4). TNM stage, age, gender, histological type, and some other clinicopathological factors were considered as prognostic factors for patients with LA-NSCLC according to previous research (5). But the predictive efficacy of these factors is poor, and more precise and comprehensive biomarkers are needed in the clinic. Immunotherapy has attracted more attention in recent years due to the rapid development of this new treatment strategy. In the phase 3 PACIFIC study of patients with unresectable, stage III NSCLC without progression after chemoradiotherapy (CRT), durvalumab [an anti-programmed death ligand 1 (PD-L1) antibody] demonstrated significant improvements versus placebo in the primary endpoints of progression-free survival (PFS) and OS (6). It suggests the important role of immune status in LA-NSCLC patients.

The tumor microenvironment (TME) is the internal environment of malignant tumor progression (7). Tumor-infiltrating immune cells are a crucial component of the TME and reflect the host antitumor immune response (8). Several studies have suggested the densities of specific tumor-infiltrating lymphocytes (TILs), such as CD3+, CD4+, and CD8+, were associated with prognosis in multiple cancers (9–11). Besides, a variety of non-lymphocyte immune factors, such as macrophages and PD-L1, were also detected in the TME that might play an important role in cancer progression (12–14). However, these studies always just analyzed the single immune feature and its potential clinical relevance. The immune status of the TME is extremely complex. One single immune feature cannot reflect the integral immune status, and its prognostic value was limited.

In gastric cancer, the TIL score evaluated by TIL intensity and percentage was found to effectively predict disease recurrence and patient survival (15). For patients with colon cancer, the immune score generated by the immune cell-related genes was proven to be the independent predictor for the patient OS, demonstrating the promising role of immune features-based signature in prognostic prediction (16). However, these studies just focused on the TIL score without the classification of subpopulation, which was hard to understand, and the functional state in the cancer immunity; or the related gene identification was derived from the gene transcriptomes from the publicly available datasets, which may affect the results in the real world. Furthermore, the role of immunoscore in LA-NSCLC remains unproven. In this study, through analyzing the expression of immune cell markers in the protein level by immunohistochemistry (IHC) assay, we aim to model an immune signature containing selected tumor-related immune features that can more informatively reflect the overall immune status. Another purpose is to construct a visualized nomogram model based on the immune signature for comprehensive individualized prediction of LA-NSCLC patients.

## Materials and Methods

### Patients

This study was carried out following the rules of the Declaration of Helsinki of 1975 and approved by the ethics committee of Shandong Cancer Hospital and Institute (Approval number: 2020008003). The screening process was shown in Supplementary Figure 1. Finally, 100 patients were retrospectively reviewed between January 2005 and January 2016. All patients signed informed consent forms and agreed to provide their pathologic specimens and clinical data for research purposes. The inclusion criteria were as follows: (1) aged 18 years or older; (2) stage III NSCLC confirmed by histopathology and radiographic results; (3) patients with neither prior therapy received concurrent CRT nor operation after assessment by a multidisciplinary team; (4) availability of follow-up data and pathologic specimens; and (5) patients without autoimmune disease or active infection such as acute gastroenteritis, appendicitis, or cholecystitis. The clinicopathologic features studied in the study included age, gender, pathological type, TNM stage, Karnofsky Performance Score (KPS), smoking index, radiotherapy technique, and radiotherapy dose. We also collected laboratory tests such as blood cell counts (lymphocyte, neutrophil, monocyte, and platelet counts) and pretreatment carcinoembryonic antigen (CEA) and hemoglobin levels.

After completion of the treatment, patients were followed every 3 months for the first 2–3 years, every 4–6 months for another 2 years, and annually thereafter. The follow-up evaluations consisted of a physical examination, complete blood count, blood biochemistry, tumor marker, thoracic computed tomography (CT) scans, abdomen B-ultrasound examination, and other examinations as needed. OS was calculated from the starting date of treatment to the date of death or censored at the date of last contact. PFS was calculated from the starting date of treatment to the date of diagnosing local recurrence/distant metastasis or to the date of last follow-up.

### Immunohistochemistry

Tumor specimens obtained by biopsy from 100 LA-NSCLC patients were fixed overnight in 10% formalin and embedded in paraffin. Serial tissue sections of 4-μm thickness were prepared for immunohistochemical stains. Briefly, the sections were deparaffinized by three incubations in xylene followed by 5-min incubations in 100, 95, and 70% ethanol and rehydrated in water. Endogenous peroxidase activity was blocked with 3% H_2_O_2_ for 5 min and then treated with 0.01 M citrate buffer pH 6.0 for 10 min in a microwave oven at 650 W. Slides were incubated with Dual Endogenous Enzyme Block (Agilent) and followed by blocking with 1% bovine serum albumin (BSA) in phosphate buffered saline (PBS)/0.1% Tween-20. Then, primary antibodies, including anti-CD3 antibody, anti-CD4 antibody, anti-CD8 antibody, anti-CD163 antibody, anti-FOX-P3 antibody, anti-PD-L1 antibody (Beijing Zhongshan Golden Bridge Biotechnology Company), and anti-Ki-67 antibody (Santa Cruz Biotechnology) at a dilution of 1:100 were used for the 1-h staining at room temperature. Real^TM^EnVision^TM^ HRP Rabbit/Mouse detection system (Dako) was used as a secondary antibody, and the sections were then counterstained with hematoxylin. Examples of low and high expression of CD3, CD4, CD8, CD163, FOX-P3, PD-L1, and Ki-67 are shown in Supplementary Figure 2.

The expression levels of all immune features were measured using a computerized software Image-Pro Plus (version 6.0, Media Cybernetics, Rockville, MD, United States). Digital images of immunohistochemically stained slides were acquired under Nikon Eclipse 80i microscopy equipped with Nikon DS-Fi1 camera. All images were taken under high magnification (400×) with consistent imaging parameters (uniform light source, exposure time, and autofocus). Integrated optical density (IOD) was used to determine the quantification of all immune features.

### Construction of Immune Signature Using the Least Absolute Shrinkage and Selection Operator Cox Regression Model

We developed an immune signature with the optimal combination of relevant features for predicting LA-NSCLC patients’ survival. To obtain a more reliable assessment of the generalizability of the signature, we applied nested repeat 10-fold cross validation, in which patients were stratified for OS events and N stage. For each fold, 90% of the patients were randomly selected as the training cohort, and the remaining 10% were used as the holdout test set. To avoid overfitting, we used the Cox regression model jointly with least absolute shrinkage and selection operator (LASSO) algorithm to select the most relevant features. Feature selection was strictly limited to the training cohort (90% of patients). The model performance was evaluated in the held-out test set using the concordance index (C-index) and the Integrated Brier Score (IBS). The entire process was repeated 100 times. Finally, in order to define a unique immune signature, we fit a Cox regression model using the most commonly selected features (>80%) during 100 repeats of cross validation. The “glmnet” package from R software (version 3.5.2) was used to perform the LASSO Cox regression model analysis.

### Statistical Analysis

The optimal cutoff value for immune signature was assessed by a time-dependent receiver operating characteristic (ROC) curve analysis and analysis of the area under the curve. The chi-square test was used to compare the relationship between immune signature and clinicopathologic features, while the Pearson correlation analysis was used to evaluate the relationship between immune signature and hematological parameters. Survival curves were generated by the Kaplan–Meier method and compared using the log-rank test. The Cox proportional hazard regression model was used to detect the independence of related factors. Variables with *P* < 0.15 in univariate analyses were entered into multivariate analyses. Results of the Cox regression modeling are presented as hazard ratios (HRs) and associated 95% confidence intervals (CIs). Variables with *P* < 0.05 were considered statistically significant.

Nomograms were established as a graphic representation of the prediction model and elaborated upon linear regression model (LRM) coefficients by using the RMS package of R (version 3.5.2). The accuracy of the predictions was evaluated by Harrell’s c-index, which was calculated *via* a bootstrap method with 1,000 resamples. The maximum value of the c-index is 1.0, indicating perfect discrimination, whereas 0.5 represents agreement by chance alone. Calibration curves were assessed graphically by plotting the observed rates against the nomogram predicted probabilities. Decision curve analysis was used to evaluate the clinical usefulness of the nomograms. To quantify the improvement of usefulness added by the immune signature, a net reclassification improvement (NRI) calculation was also applied (17). All statistical analyses were conducted using R 3.5.2 software (Institute for Statistics and Mathematics, Vienna, Austria) and SPSS 23.0 (SPSS Inc., Chicago, IL, United States).

## Results

### Baseline Characteristics of Locally Advanced Non-small-Cell Lung Cancer Patients

A total of 100 patients with inoperable LA-NSCLC were enrolled in the study. The median follow-up time was 36.3 months (1.9–115.3 months). Table 1 shows the clinicopathological characteristics of these patients. Among them, male patients accounted for the majority (78%), 51% of the patients had squamous cell carcinoma, and most patients received 3D-CRT radiotherapy (55%).

**TABLE 1:** The clinicopathological characteristics of inoperable LA-NSCLC patients.

Characteristic	All patients (*N* = 100)	
	*N*	%
**Age (years)**		
<60	50	50.0
≥60	50	50.0
**Sex**		
Male	78	78.0
Female	22	22.0
**Histology subtype**		
SCC	51	51.0
Non-SCC	49	49.0
**Stage**		
IIIA	45	45.0
IIIB	55	55.0
**Smoking index**		
<600	49	49.0
≥600	51	51.0
**KPS**		
≤80	58	58.0
>80	42	42.0
**Radiotherapy technique**		
3D-CRT	55	55.0
IMRT	45	45.0
Radiotherapy dose (Gy)		
≤60	60	60.0
>60	40	40.0
**Pretreatment hemoglobin (g/L)**		
≤130	47	47.0
>130	53	53.0
**Pretreatment CEA (ng/ml)**		
≤4.4	42	42.0
>4.4	58	58.0

*CEA, carcinoembryonic antigen; CRT, chemoradiotherapy; IMRT, intensity modulated radiotherapy; KPS, Karnofsky Performance Score; LA-NSCLC, locally advanced non-small-cell lung cancer; SCC, squamous cell carcinoma.*

### Construction of Immune Signature

In the holdout test sets, the mean C-index was 0.619 (*t*-test *P* < 0.001), and the mean IBS was 0.033 for 100 repeats of 10-fold cross validation. The selection of the immuno-features is shown in Supplementary Figure 3. Finally, five model parameters were screened out in seven independent tumor-related immune features, including CD8, CD163, FOXP3, PD-L1, and Ki-67 (Supplementary Figure 4). Based on these five parameters, a Cox proportional hazard model with OS as the endpoint was established for immune signature. The formula for calculating immune signature is: Immune-score = 0.00001233048 × CD8 + 0.000002998373 × CD163 + 0.000009072303 × PD-L1 – 0.0000288154 × FOXP3 – 0.000001788912 × Ki-67.

The correlation between immune signature and clinicopathological factors was further analyzed. As shown in Supplementary Table 1, the immune signature was not associated with all the clinical and hematological parameters, demonstrating that it was independent of the clinicopathological factors (Supplementary Figure 5).

### Prognostic Effects of Immune Signature

**FIGURE 1:**
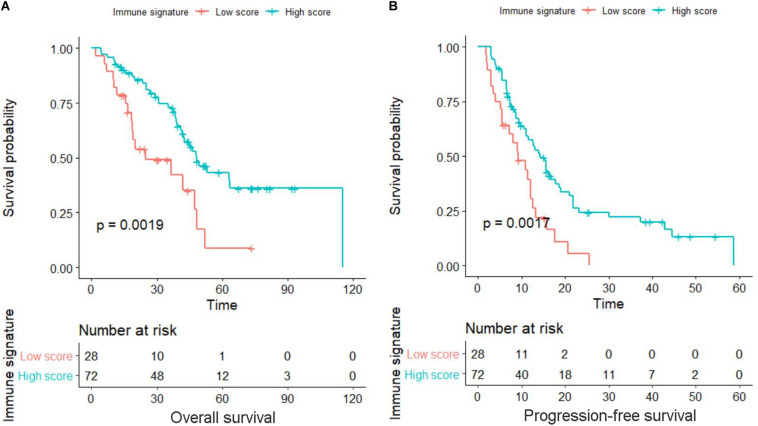
Kaplan–Meier survival curves in locally advanced non-small-cell lung cancer (LA-NSCLC) patients according to immune signature-defined high-score and low-score groups. **(A)** Survival curve for overall survival (OS). **(B)** Survival curve for progression-free survival (PFS). *P*-values were calculated using the log-rank test.

Receiver operating characteristic analysis with OS as the endpoint was used to determine the best cutoff value for the immune signature, which was calculated as −0.05. Accordingly, all patients were divided into a low-immune signature group (immune signature <−0.05) and a high-immune signature group (immune signature ≥−0.05). As shown in Figure 1, the OS and PFS of the high-immune signature group are significantly better than those of the low-immune signature group (*P* = 0.002 and *P* = 0.002, respectively). The 2-year and 5-year OS rates for the high-immune signature group were 82.5 and 39.8%, respectively, while for the low-immune signature group were only 49.2 and 8.8%, respectively. Similarly, the 1-year and 2-year PFS rates for the high-immune signature group were 56.0 and 22.2%, respectively, while for the low-immune signature group were only 30.7 and 5.5%, respectively.

**FIGURE 2:**
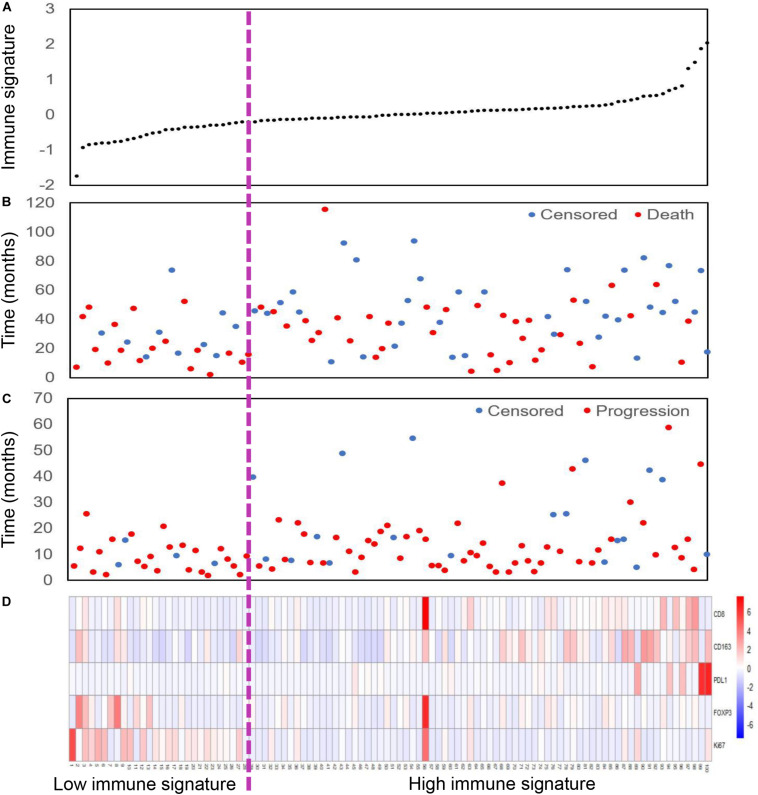
Immune signature analysis of inoperable locally advanced non-small-cell lung cancer (LA-NSCLC) patients (*n* = 100). **(A)** Immune signature distribution. **(B)** Survival status of inoperable LA-NSCLC patients. **(C)** Recurrence status of inoperable LA-NSCLC patients. **(D)** Color-gram of the expression profiles of five immune features in inoperable LA-NSCLC patients. Rows represent five immune features, and columns represent patients. Magenta dotted line represents the immune signature cutoff dividing the patients into high- and low-immune signature groups.

We also assessed the distribution of immune signature, recurrence and survival status, and the expression of the five selected tumor-related immune features. Patients with low-immune signature were more likely to have recurrence and death (Figure 2).

### Immune Signature-Based Nomogram for Locally Advanced Non-small-Cell Lung Cancer Patients

**FIGURE 3:**
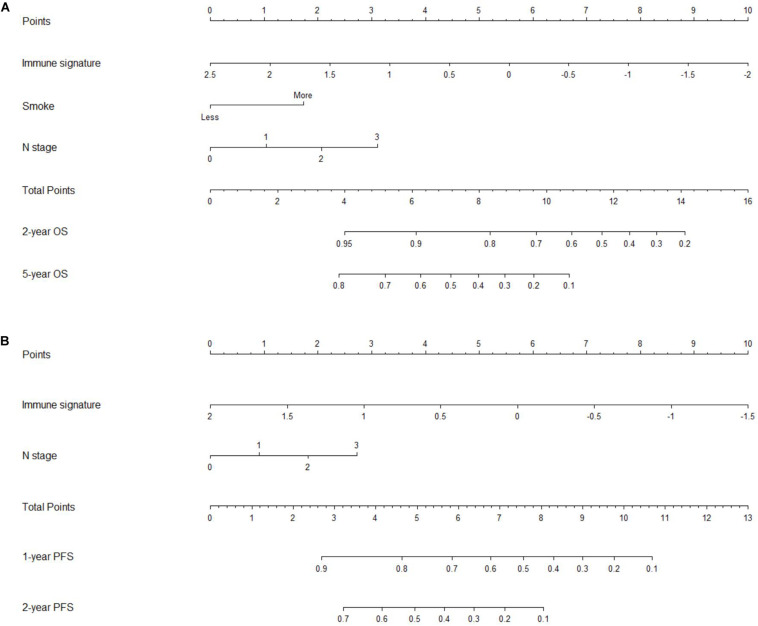
Nomograms for predicting the overall survival (OS) and progression-free survival (PFS) of patients with inoperable locally advanced non-small-cell lung cancer (LA-NSCLC). **(A)** Nomogram showed the results of prognostic models using immune signature and clinicopathological characteristics for 2- and 5-year OS. **(B)** Nomogram showed the results of prognostic models using immune signature and clinicopathological characteristics for 1- and 2-year PFS.

**FIGURE 4:**
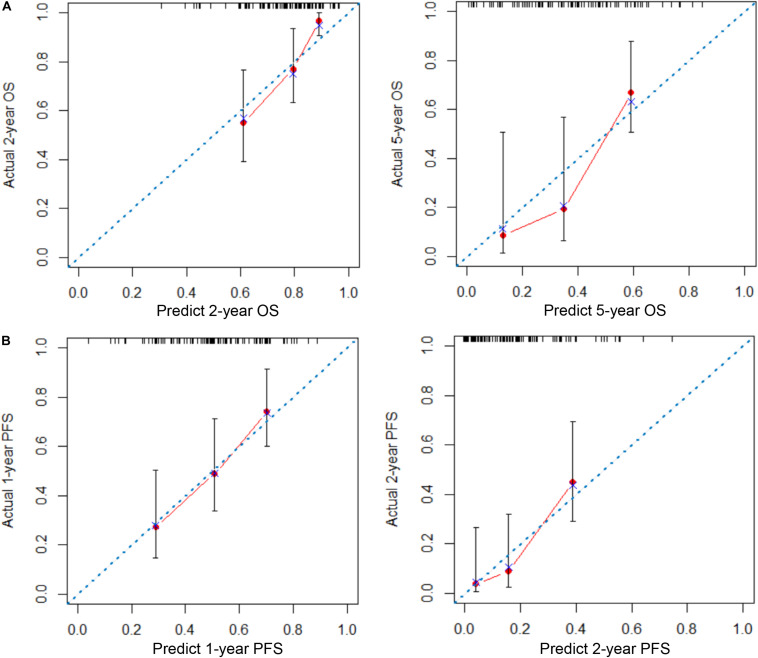
Calibration plots of nomograms for predicting overall survival (OS) and progression-free survival (PFS) in patients with inoperable locally advanced non-small-cell lung cancer (LA-NSCLC). **(A)** Calibration plots for predicting 2- and 5-year OS. **(B)** Calibration plots for predicting 1- and 2-year PFS. The *x*-axis is the nomogram-predicted probability of survival, and the *y*-axis is actual survival. The reference line is 45° and indicates perfect calibration.

To provide clinicians with a quantitative method to predict the 2- and 5-year OS and 1- and 2-year PFS probabilities of inoperable LA-NSCLC patients, we established an immune signature-based nomogram containing immune signature and clinicopathologic features. Independent prognostic factors identified through multivariate Cox regression model were included in the immune signature-based nomogram. Among them, immune signature (HR = 2.080; 95% CI 1.040–4.161; *P* = 0.038), N stage (HR = 1.937; 95% CI 1.037–3.617; *P* = 0.030), and smoking status (HR = 1.435; 95% CI 1.036–1.987; *P* = 0.038) were verified to be independent prognostic factors for OS. Immune signature (HR = 1.760; 95% CI 1.110–2.792; *P* = 0.016) and N stage (HR = 1.354; 95% CI 1.064–1.722; *P* = 0.014) were independent factors for PFS (Table 2). These predictive factors were then further integrated into the prognostic nomograms for OS and PFS (Figures 3A,B), and their C-indexes are 0.692 (95% CI: 0.645–0.739) and 0.672 (95% CI: 0.637–0.707), respectively. Moreover, the calibration curves showed good agreement between the prediction by immune signature-based nomograms and actual observation (Figure 4).

**TABLE 2:** Univariate and multivariate survival analyses of OS and PFS in patients with inoperable LA-NSCLC.

	OS						PFS					
	Univariate analysis			Multivariate analysis			Univariate analysis			Multivariate analysis		
	HR	95% CI	*P*-value	HR	95% CI	*P*-value	HR	95% CI	*P*-value	HR	95% CI	*P*-value
Age	0.902	0.526–1.546	0.708				0.840	0.534–1.323	0.452			
Sex	0.476	0.229–0.991	0.047				0.901	0.525–1.548	0.706			
Histology	1.062	0.608–1.855	0.832				0.878	0.553–1.394	0.581			
T stage	0.721	0.523–0.996	0.047				0.986	0.785–1.237	0.901			
N stage	1.598	1.139–2.242	0.007	1.435	1.036–1.987	0.030	1.398	1.090–1.794	0.008	1.354	1.064–1.722	0.014
Smoking index	2.359	1.344–4.141	0.003	1.937	1.037–3.617	0.038	1.242	0.788–1.959	0.351			
Consolidation chemotherapy	1.562	0.900–2.714	0.113				1.025	0.627–1.677	0.921			
KPS	0.808	0.459–1.422	0.460				0.842	0.527–1.345	0.472			
Radiotherapy dose	1.730	1.013–3.031	0.046				1.493	0.929–2.398	0.098			
Radiotherapy technique	1.596	0.923–2.761	0.095				1.049	0.661–1.663	0.840			
Pretreatment hemoglobin	1.042	0.596–1.821	0.886				1.019	0.644–1.612	0.936			
Pretreatment CEA	1.576	0.844–2.942	0.153				1.384	0.836–2.293	0.206			
Immune signature	0.367	0.195–0.693	0.002	0.481	0.240–0.961	0.038	0.548	0.340–0.885	0.014	0.568	0.358–0.901	0.016

*CEA, carcinoembryonic antigen; HR, hazard ratio; KPS, Karnofsky Performance Score; LA-NSCLC, locally advanced non-small-cell lung cancer; OS, overall survival; PFS, progression-free survival.*

### Assessment of Incremental Value of Immune Signature in Individual Overall Survival and Progression-Free Survival Performance

**FIGURE 5:**
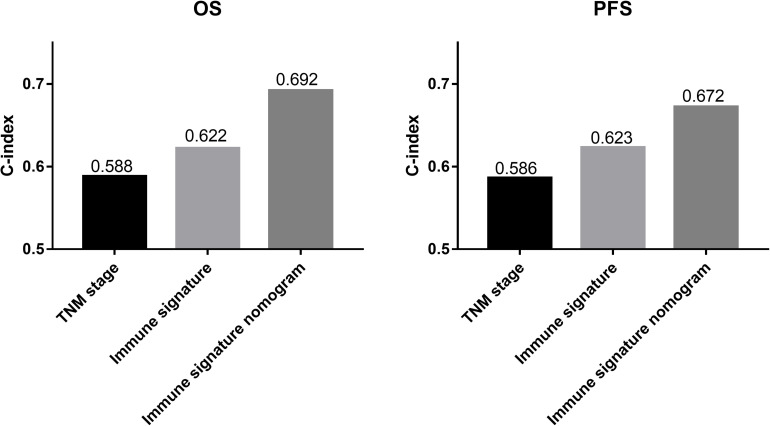
The histograms represent the C-index of different prediction models for patients with inoperable locally advanced non-small-cell lung cancer (LA-NSCLC). The predictive performance of the immune signature nomograms and immune signatures were better than that of the TNM staging system for overall survival (OS) and progression-free survival (PFS).

**FIGURE 6:**
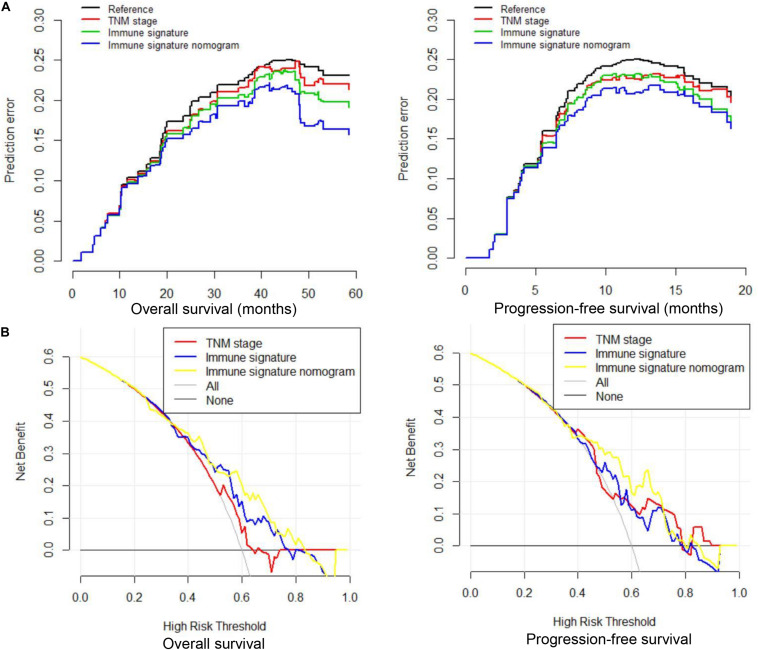
Prediction error curves and decision curve analysis for each model in inoperable locally advanced non-small-cell lung cancer (LA-NSCLC) patients. **(A)** Prediction error curves for overall survival (OS) and progression-free survival (PFS); lower prediction errors indicate higher model accuracy. **(B)** Decision curve analysis for OS and PFS. The *y*-axis measures the net benefit. The net benefit was calculated by summing the benefits (true-positive results) and subtracting the harms (false-positive results), weighting the latter by a factor related to the relative harm of an undetected cancer compared with the harm of unnecessary treatment.

We compared the predictive ability of the TNM staging system, the immune signature, and the immune signature-based nomogram. In terms of C-index, the immune signature and immune signature-based nomogram showed better discrimination in both OS and PFS compared to the TNM staging system (OS: 0.622 vs. 0.692 vs. 0.588; PFS: 0.623 vs. 0.672 vs. 0.586, respectively) (Figure 5). Similarly, immune signature (OS: 2.62%; PFS: 7.95%) and immune signature-based nomogram (OS: 32.96%; PFS: 9.22%) also showed significant improvement in the net classification (Supplementary Figure 6). The prediction error curves for all models are shown in Figure 6A. For both OS and PFS, the immune signature-based nomogram had the lowest prediction error rate, while the TNM staging system had the highest prediction error rate. In clinical application, the decision curve analysis showed that the immune signature-based nomogram had a higher net benefit than the TNM staging system and immune signature in most reasonable thresholds, especially for the threshold probability of 0.6–0.8 for OS and 0.5–0.7 for PFS (Figure 6B).

## Discussion

Accurate prognostic assessment is critical for the follow-up treatment of LA-NSCLC patients. In routine clinical practice, the TNM staging system is commonly used by oncologists and patients as a determining prognostic factor. However, the heterogeneity of LA-NSCLC is very large, and the clinical outcomes of these patients with the same cancer stage are different, indicating that the current staging system is not sufficient to accurately assess the prognosis (4). In this study, we constructed a classifier called immune signature that contains five immuno-features. Independent of TNM staging, the immune signature is a novel prognostic tool for assisted oncologists to identify clinical outcomes in patients with LA-NSCLC. Different from the single immune indicators mentioned in the previous study, our immune signature was constructed by the LASSO Cox regression model, which can significantly improve its prediction accuracy (18, 19). In addition, C-indexes showed that the prognostic value of the combing nomogram integrated with immune signature is significantly better than that of the TNM staging system, which also suggests immune signature reinforced the prognostic ability of TNM stage, thereby adding prognostic value to the TNM staging system.

Cancer progression is determined not only by the malignant behavior of tumors but also by the immune microenvironment. In recent years, immune cell markers such as CD8+, CD163+, PD-L1+, and FOXP3 have shown prognostic impacts in patients suffering from solid tumors including NSCLC (9, 10, 20). However, a single immune feature cannot reflect the overall immune status of patients (21). In this study, we first selected a series of immune markers commonly reported in clinical studies for immunohistochemical analysis. Then, five of these immune features were identified using nested repeat 10-fold cross validation and LASSO analysis to construct one immune signature that could comprehensively reflect the immune status. Lambda value with the smallest cross-validation error was selected to maximize the accuracy of the immune signature on the premise of avoiding overfitting (22, 23). It ensured that immune signature consisting of five features is the best choice in comprehensive consideration of overfitting and prediction accuracy. In addition, our findings demonstrated that this immune signature was highly correlated with PFS and OS (*P* = 0.002 and *P* = 0.002, respectively).

Recently, the concept of immune signature was introduced into large clinical trials and research. Jiang et al. (24) developed an ImmunoScore signature containing CD3, CD8, CD45, and CD66. The ImmunoScore classifier could effectively predict recurrence and survival of gastric cancer and was a useful predictive tool to identify stage II and III gastric cancer patients who would benefit from adjuvant chemotherapy (24). Furthermore, IMpower150 is the first randomized phase 3 trial evaluating atezolizumab + chemo [carboplatin (C) + paclitaxel (P)] ± bevacizumab vs. CP + bevacizumab in first-line non-squamous NSCLC (25). In this clinical trial, Teff immune signature [including mRNA expression levels of PD-L1, chemokine ligand 9 (CXCL9), and interferon-γ (IFN-γ) genes] was analyzed as an exploratory biomarker. In Teff-high patients, group B (Atezo + CP + Bev) significantly prolonged PFS compared with group C (CP + Bev), with mPFS of 11.3 vs. 6.8 months (HR = 0.505; 95% CI: 0.377–0.675; *P* < 0.0001). The Teff immune signature is thus considered to be a surrogate for PD-L1 expression, and its practicality in clinical practice is promising.

Our study for the first time demonstrated the prognostic value of immune signature in LA-NSCLC, which may provide more information on the immune status of the tumor and the TME. A large number of studies have shown that the preexisting tumoral and peritumoral immune infiltration is related to the efficacy of immune checkpoint inhibitors (26–28). Hegde et al. (29) proposed that tumors can be divided into three different immune phenotypes: immune-inflamed, immune-excluded, and immune-desert. Immune-inflamed phenotype is characterized by diffuse infiltration of T lymphocytes, expression of cell checkpoint markers (such as PD-L1), and a high mutational burden, which is always sensitive to immunotherapy. Immune-excluded phenotype is characterized by the activation of angiogenesis and other pathways, which limits the infiltration of lymphocytes into the tumors. And immune-desert phenotype is characterized by decreased infiltration of lymphocytes. As durvalumab (an anti-PD-L1 antibody) has been reported to be effective in patients with LA-NSCLC after concurrent CRT, our immune signature may have great potential for identifying patients who are suitable for subsequent immunotherapy, or at least provide a promising reference for the screening of the useful biomarker predicting the prognosis of LA-NSCLC receiving immunotherapy.

In our immune signature, CD8, CD163, FOXP3, PD-L1, and Ki-67 were recognized as valid immune features. Among them, CD8 played a positive regulatory role in immune signature, consistent with previous studies that demonstrated that CD8 + TILs were associated with better prognosis in NSCLC patients (9). Similarly, our study found that PD-L1 and CD163 also played positive regulatory roles in immune signature. Although the relationship between PD-L1 expression and prognosis was still controversial, its positive role may be associated with its essential modulation for effector T cells to survive during the contraction phase of the immune response and the direct function in overcoming the immunosuppressive TME (30). CD163 is a marker of M2 type tumor-associated macrophage (TAM). Biologically, the M1 and M2 subpopulations of macrophages are expected to associate with inverse antitumoral or pro-tumoral functions, respectively. However, we and other researchers have observed that the relationship between M1 and M2 subtype infiltration and survival outcomes varied in different studies (31, 32). A reasonable explanation is the mutual transformation of M1 and M2 TAM (33). Besides, Kim et al. (32) speculated that M2 TAM is closely related to lymphocyte infiltration. It is involved in the effective recruitment process of lymphocytes and cooperates with helper T cells/cytotoxic cells to induce an antitumor immune response (32). These mechanisms may be the biological basis of M2 TAM as a positive prognostic factor in our study. In contrast, FOXP3 and Ki-67 were shown to perform negative roles in immune signature. FOXP3, a surface marker of regulatory T cell (Treg), could promote cancer cells to escape immune surveillance (34). It was not surprising that high infiltration of Tregs in the TME was associated with poor prognosis in patients with LA-NSCLC. Ki-67, a widely used biomarker for tumor proliferation (35), was also determined to be a poor prognostic factor in our present study, confirming its critical role in the prediction of tumor patient prognosis.

Another contribution of our research was that we developed nomogram models that could amplify the clinical value of immune signature. Our nomograms contain all independent prognostic factors identified in multivariate analysis. The results showed that patients with a lower immune signature, smoke more, and have a higher N stage have worse prognosis. Besides, it was indicated that the accuracy of immune signature nomogram was better than the traditional TNM staging system in terms of discriminating ability (OS: 0.692 vs. 0.588; PFS: 0.672 vs. 0.586, respectively) or net weight classification (OS: 32.96%; PFS: 9.22%), suggesting that immune signature plays a complementary role in improving the prognostic value. Calibration plots showed that the 2- and 5-year OS rate and 1- and 2-year PFS rate predicted by our nomograms closely match the actual survival rate estimated by the Kaplan–Meier method, and the prediction error curve also confirmed that our immune signature nomogram had a low prediction error rate. Compared with other prognostic models of LA-NSCLC reported by previous studies, our immune signature nomogram comprehensively analyzed information on TIL features, macrophage infiltration, tumor proliferation, and clinicopathological factors, which could more accurately reflect the characteristics of patients with LA-NSCLC from facets of both tumor biology and patient general status (36–38).

This study has several limitations. First, it was a retrospective study with all specimens obtained from patients in China prior to durvalumab introduction in LA-NSCLC treatment, and the spatial and temporal representations are limited to a certain extent. Second, the immune characteristics analyzed by IHC assay in our study were limited due to the less tumor tissue mostly obtained by fine-needle aspiration. Third, we retrospectively collected specimens from 2005 to 2016. The freshness of the specimens may affect the accuracy of the IHC analysis, as the expression of PD-L1 decreases with the age of the specimens. In the future, the microarray analysis may be performed in the prospective trials, which is helpful for providing more information about the immune status of the tumor environment. Nevertheless, we for the first time constructed the immune signature in patients with LA-NSCLC based on the commonly used immune features. Besides, this finding might provide helpful reference for identifying the suitable patient with LA-NSCLC receiving immunotherapy.

## Conclusion

Our study demonstrated that immune signature based on multiple immune features could effectively predict OS and PFS for inoperable LA-NSCLC patients. Furthermore, the immune signature nomogram incorporating immuno-signature is significantly better than the traditional TNM staging system in prediction efficiency and accuracy. It may be a new LA-NSCLC assessment model that will facilitate patient counseling, decision-making regarding individualized therapy, and follow-up scheduling in the future.

## Data Availability Statement

The raw data supporting the conclusion of this article will be made available by the authors, without undue reservation.

## Ethics Statement

The studies involving human participants were reviewed and approved by the Ethics Committee of Shandong Cancer Hospital and Institute (Shandong, China). The patients/participants provided their written informed consent to participate in this study.

## Author Contributions

LW and XS contributed to the study concept and design, and critical revision of the manuscript for important intellectual content. MG performed the data analysis and drafted the manuscript. WL, BL, SW, BZ, and BF contributed to data collection and interpretation. All authors contributed to the revision of the manuscript, and read and approved the final manuscript.

## Conflict of Interest

The authors declare that the research was conducted in the absence of any commercial or financial relationships that could be construed as a potential conflict of interest.

## Supplementary Material

Supplementary Figure 1 Screening process and treatment process of patients included in this study.

Supplementary Figure 2 Examples of low and high expression of CD3, CD4, CD8, CD163, FOX-P3, PD-L1, and Ki-67.

Supplementary Figure 3 The selection of immune-features through 100 repetitions of 10-fold cross validation.

Supplementary Figure 4 Immune feature selection using the least absolute shrinkage and selection operator (LASSO) Cox regression model. **(A)** LASSO coefficient profiles of the seven immune features. **(B)** Tuning parameter (λ) selection in the LASSO model used 10-fold cross validation *via* minimum criteria.

Supplementary Figure 5 The correlation between immune signature and hematological parameters in inoperable locally advanced non-small-cell lung cancer (LA-NSCLC) patients. **(A)** Correlation between immune signature and pre-chemoradiotherapy hematological parameters. **(B)** Correlation between immune signature and post-chemoradiotherapy hematological parameters. The size of each point represents the correlation coefficient.

Supplementary Figure 6 Net reclassification improvement (NRI) of immune signature and immune signature nomogram compared to TNM staging system in inoperable locally advanced non-small-cell lung cancer (LA-NSCLC) patients. **(A)** NRI for overall survival (OS). **(B)** NRI for progression-free survival (PFS).

Supplementary Table 1 The relationship between immune signature and clinicopathological features in operable LA-NSCLC patients. CEA, carcinoembryonic antigen; CRT, chemoradiotherapy; KPS, Karnofsky Performance Score; LA-NSCLC, locally advanced non-small-cell lung cancer.
