# Supplementary figures and images for: Multiple Immune Features-Based Signature for Predicting Recurrence and Survival of Inoperable LA-NSCLC Patients
Source: Front Oncol. 2020 Oct 14;10:571380. doi: 10.3389/fonc.2020.571380 (PMC7591766; doi:10.3389/fonc.2020.571380)

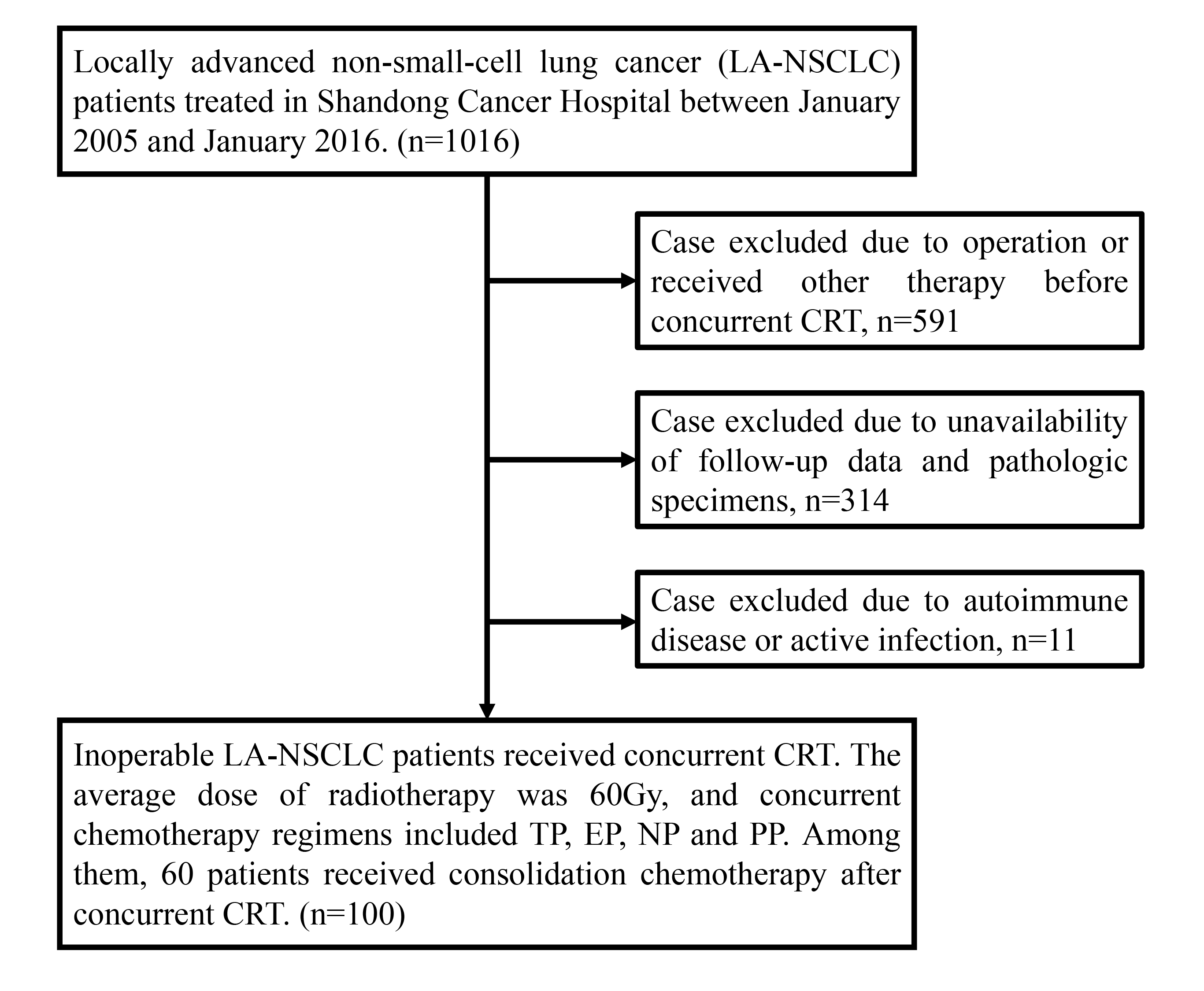

Supplement: Supplementary Figure 1 — Screening process and treatment process of patients included in this study. [file Image_1.tif]

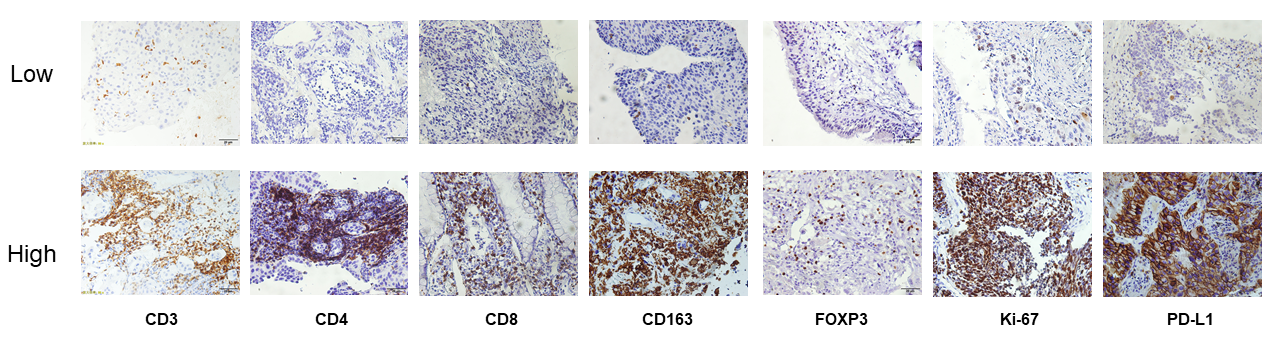

Supplement: Supplementary Figure 2 — Examples of low and high expression of CD3, CD4, CD8, CD163, FOX-P3, PD-L1, and Ki-67. [file Image_2.tif]

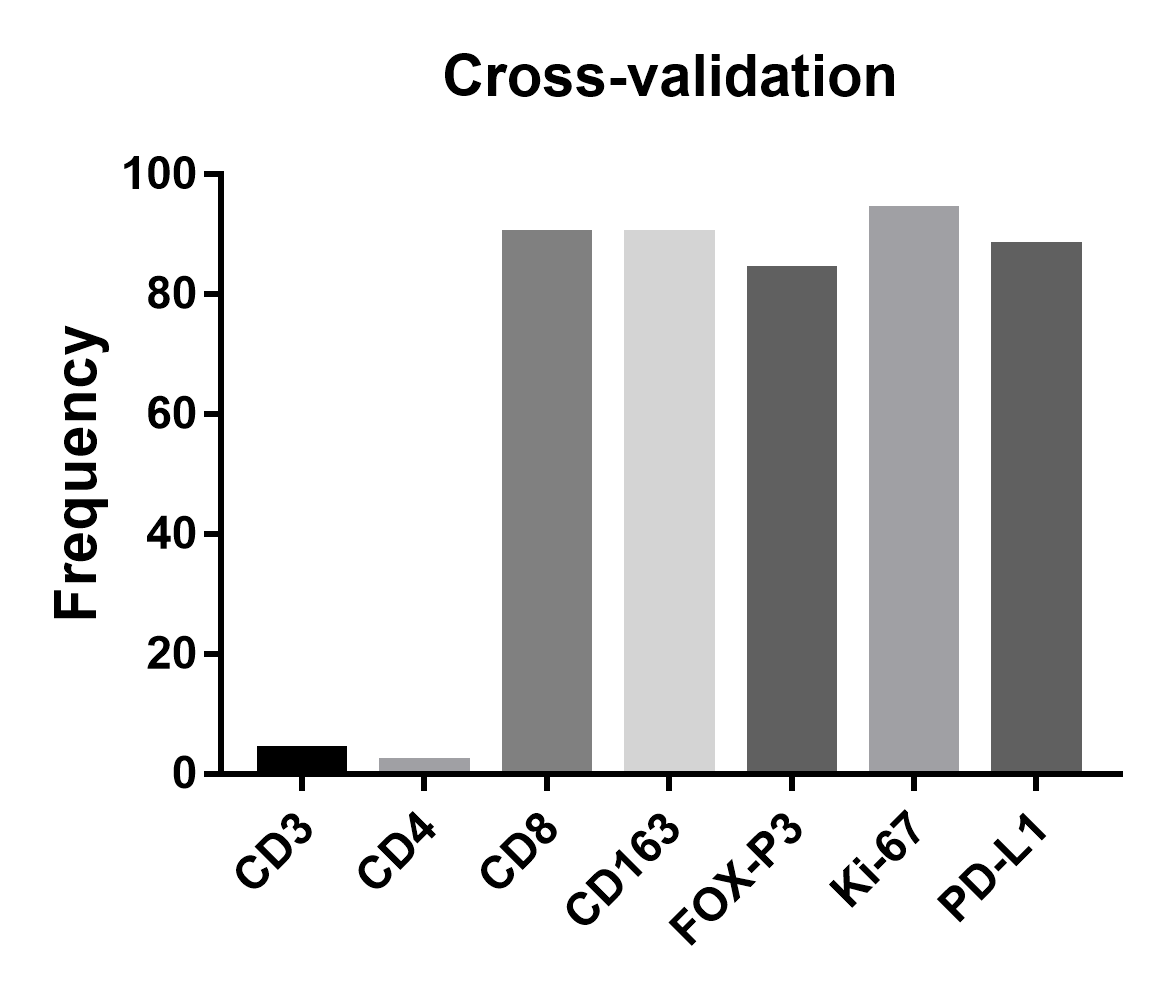

Supplement: Supplementary Figure 3 — The selection of immune-features through 100 repetitions of 10-fold cross validation. [file Image_3.tif]

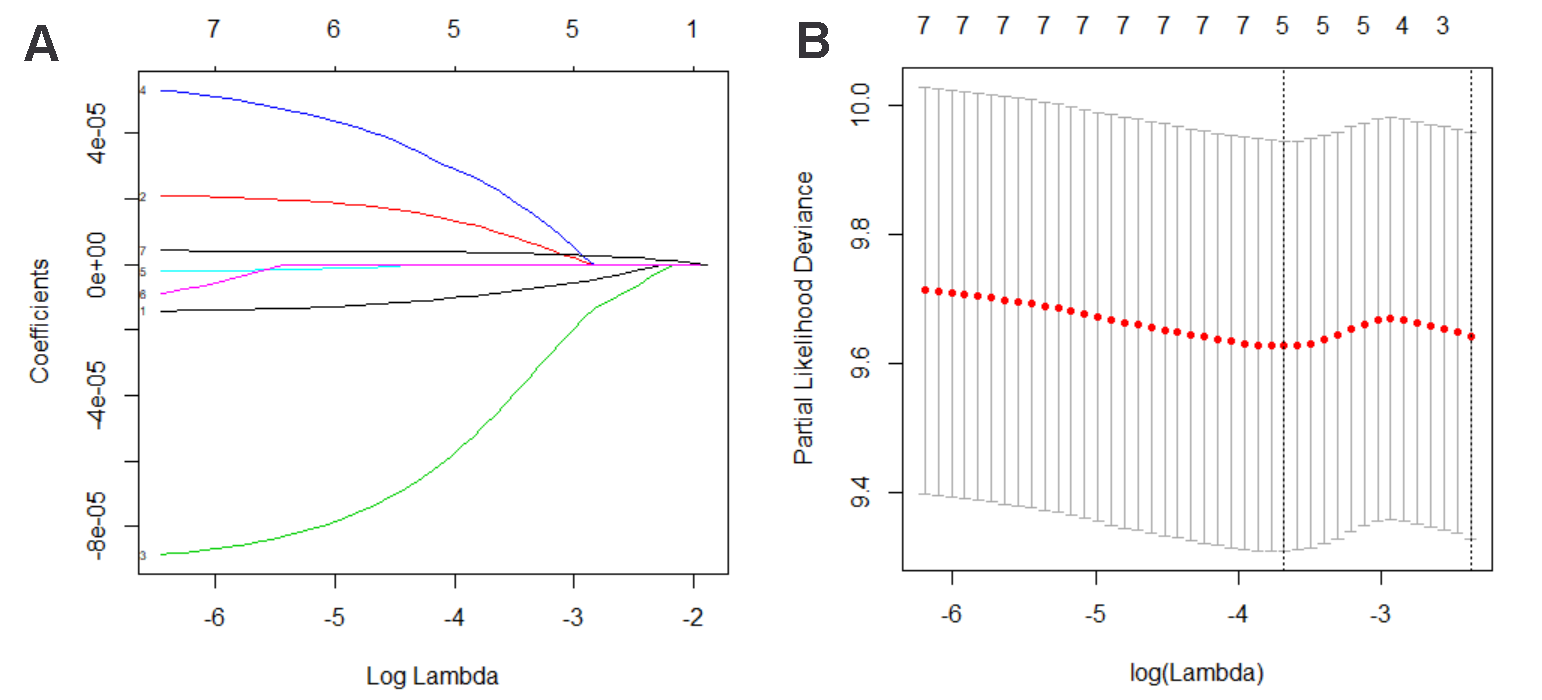

Supplement: Supplementary Figure 4 — Immune feature selection using the least absolute shrinkage and selection operator (LASSO) Cox regression model. (A) LASSO coefficient profiles of the seven immune features. (B) Tuning parameter (λ) selection in the LASSO model used 10-fold cross validation via minimum criteria. [file Image_4.tif]

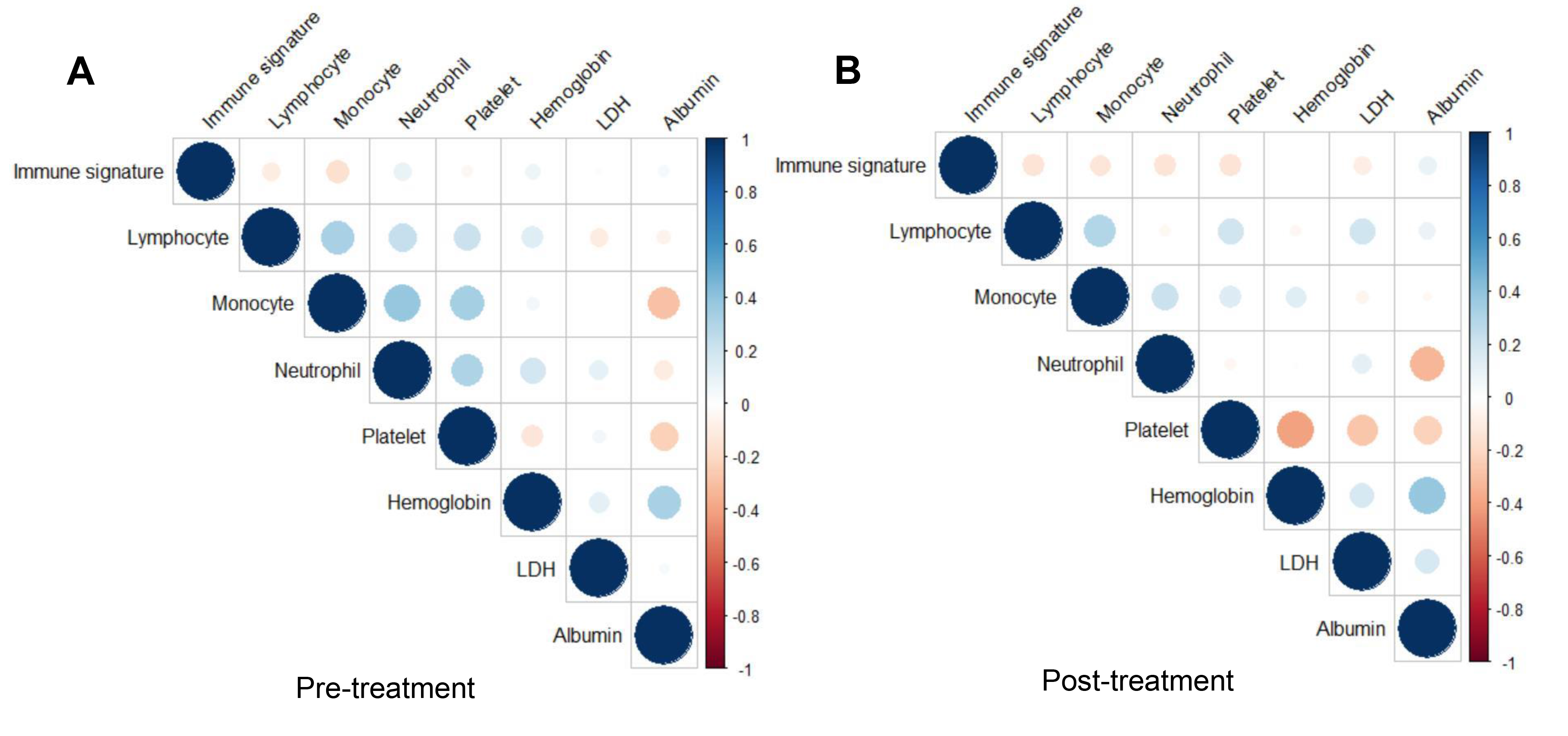

Supplement: Supplementary Figure 5 — The correlation between immune signature and hematological parameters in inoperable locally advanced non-small-cell lung cancer (LA-NSCLC) patients. (A) Correlation between immune signature and pre-chemoradiotherapy hematological parameters. (B) Correlation between immune signature and post-chemoradiotherapy hematological parameters. The size of each point represents the correlation coefficient. [file Image_5.tif]

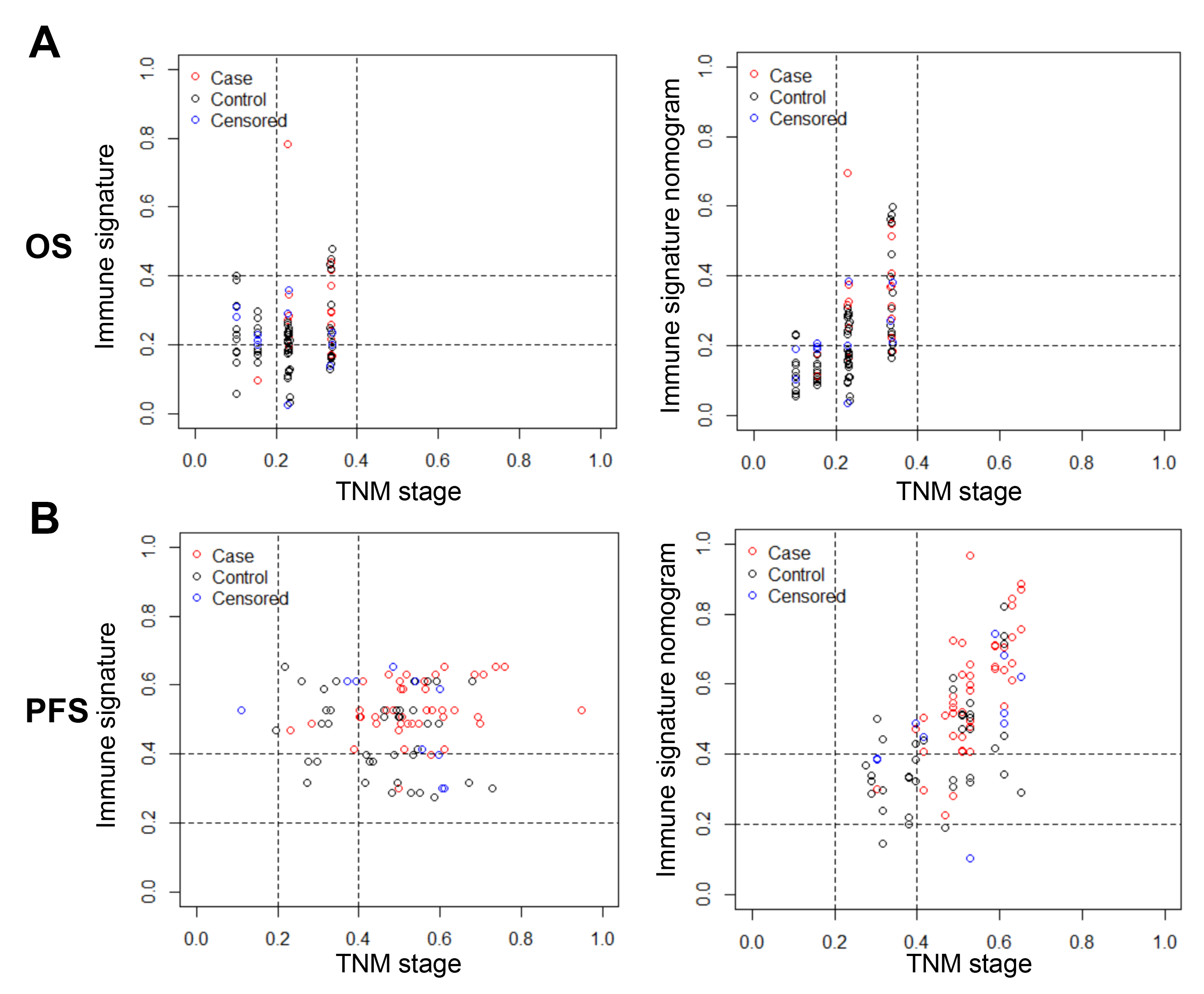

Supplement: Supplementary Figure 6 — Net reclassification improvement (NRI) of immune signature and immune signature nomogram compared to TNM staging system in inoperable locally advanced non-small-cell lung cancer (LA-NSCLC) patients. (A) NRI for overall survival (OS). (B) NRI for progression-free survival (PFS). [file Image_6.tif]
