# Supplementary material for: Multiple Immune Features-Based Signature for Predicting Recurrence and Survival of Inoperable LA-NSCLC Patients
Source: Front Oncol. 2020 Oct 14;10:571380. doi: 10.3389/fonc.2020.571380 (PMC7591766; doi:10.3389/fonc.2020.571380)
Supplement: Supplementary Table 1 — The relationship between immune signature and clinicopathological features in operable LA-NSCLC patients. CEA, carcinoembryonic antigen; CRT, chemoradiotherapy; KPS, Karnofsky Performance Score; LA-NSCLC, locally advanced non-small-cell lung cancer. [file Table_1.docx]

**Supplementary Table 1** The relationship between immune signature and clinicopathological features in operable LA-NSCLC patients.

|  | **Immune-score** | | | | |  |
| --- | --- | --- | --- | --- | --- | --- |
|  | **Low** | |  | **High** | |  |
|  | **No.** | **%** |  | **No.** | **%** | **P** |
| Age(years) |  |  |  |  |  | 0.824 |
| ＜60 | 35 | 48.6 |  | 15 | 53.6 |  |
| ≥60 | 37 | 51.4 |  | 13 | 46.4 |  |
| Sex |  |  |  |  |  | 0.602 |
| Male | 55 | 76.4 |  | 23 | 82.1 |  |
| Female | 17 | 23.6 |  | 5 | 17.9 |  |
| Histology subtype |  |  |  |  |  | 0.825 |
| SCC | 36 | 50.0 |  | 15 | 53.6 |  |
| Non-SCC | 36 | 50.0 |  | 13 | 46.4 |  |
| T stage |  |  |  |  |  | 0.752 |
| 1 | 10 | 13.9 |  | 2 | 7.1 |  |
| 2 | 25 | 34.7 |  | 9 | 32.1 |  |
| 3 | 16 | 22.2 |  | 8 | 28.6 |  |
| 4 | 21 | 29.2 |  | 9 | 32.1 |  |
| N stage |  |  |  |  |  | 0.058 |
| 0 | 7 | 9.7 |  | 5 | 17.9 |  |
| 1 | 14 | 19.4 |  | 2 | 7.1 |  |
| 2 | 33 | 45.8 |  | 8 | 28.6 |  |
| 3 | 18 | 25.0 |  | 13 | 46.4 |  |
| Smoking index |  |  |  |  |  | 0.121 |
| ＜600 | 39 | 54.2 |  | 10 | 35.7 |  |
| ≥600 | 33 | 45.8 |  | 18 | 64.3 |  |
| KPS |  |  |  |  |  | 0.262 |
| ≤80 | 39 | 54.2 |  | 19 | 67.9 |  |
| ＞80 | 33 | 45.8 |  | 9 | 32.1 |  |
| Radiotherapy technique |  |  |  |  |  | 0.179 |
| 3D-CRT | 43 | 59.7 |  | 12 | 42.9 |  |
| IMRT | 29 | 40.3 |  | 16 | 57.1 |  |
| Radiotherapy dose (Gy) |  |  |  |  |  | 0.654 |
| ≤60 | 42 | 58.3 |  | 18 | 64.3 |  |
| ＞60 | 30 | 41.7 |  | 10 | 35.7 |  |
| Pretreatment hemoglobin (g/L) | |  |  |  |  | 0.118 |
| ≤130 | 30 | 41.7 |  | 17 | 60.7 |  |
| ＞130 | 42 | 58.3 |  | 11 | 39.3 |  |
| Pretreatment CEA (ng/ml) |  |  |  |  |  | 0.262 |
| ≤4.4 | 33 | 45.8 |  | 9 | 32.1 |  |
| ＞4.4 | 39 | 54.2 |  | 19 | 67.9 |  |

CEA, carcinoembryonic antigen; CRT, chemoradiotherapy; KPS, Karnofsky Performance Score; IMRT, intensity modulated radiotherapy; LA-NSCLC, locally advanced non-small-cell lung cancer; SCC, squamous cell carcinoma.
